# Polyculture of Juvenile Dog Conch *Laevistrombus canarium* Reveals High Potentiality in Integrated Multitrophic Aquaculture (IMTA)

**DOI:** 10.3390/biology10080812

**Published:** 2021-08-22

**Authors:** Yung-Cheng Chang, Chia-Huan Ma, Hung-Tai Lee, Te-Hua Hsu

**Affiliations:** 1 Department of Aquaculture, National Taiwan Ocean University, Keelung 20224, Taiwan; corry0826@gmail.com (Y.-C.C.); booshhoh@gmail.com (C.-H.M.); 2 Center of Excellence for the Oceans, National Taiwan Ocean University, Keelung 20224, Taiwan; 3 Department of Environmental Biology and Fisheries Science, National Taiwan Ocean University, Keelung 20224, Taiwan; hungtailee@gmail.com

**Keywords:** hatchery, water temperature, salinity, coculture, diatom, breeding

## Abstract

**Simple Summary:**

The dog conch (*Laevistrombus canarium*) is a marine gastropod mollusk widely distributed in the Indo-Pacific region. It is an economically crucial species; however, its population has been declining due to overfishing and overexploitation. Hence, we developed a novel polyculture and water-flow method for mass production of this species. Furthermore, the findings from this work also uncover the potentiality of *L. canarium* in integrated multitrophic aquaculture (IMTA) and its implication for aquaculture and resource restoration.

**Abstract:**

*Laevistrombus canarium*, also known as dog conch, is a marine gastropod mollusk widely distributed in the Indo-Pacific region. It is an economically crucial species; however, its population has been declining due to overfishing and overexploitation. In this study, the suitable salinity for juvenile *L. canarium* was between 20 and 35‰. Diatoms and biological detritus by using flow-water from the fish pool were the most favorable diets for newly metamorphosed and 10 mm juveniles. In the polyculture experiment, *L. canarium* was cultured with whiteleg shrimp, tilapia, small abalone, purple sea urchin, and collector urchin. Better growth was found in all co-culture groups except with whiteleg shrimp. We also found that the polyculture system with or without substrates significantly affected the growth of juveniles. Additionally, we observed that water temperature was the most crucial factor for growth and survival; a water temperature of less than 10 °C might cause the death of *L. canarium*. We have proposed a novel polyculture and water-flow method for mass production of *L. canarium* and evaluated the feasibility and benefits of polyculture with other species. The findings from this work reveal the potentiality of *L. canarium* in integrated multitrophic aquaculture (IMTA) and its implication for aquaculture and resource restoration.

## 1. Introduction

*Laevistrombus canarium*, commonly known as dog conch, is a marine gastropod mollusk belonging to the family Strombidae. It is widely distributed in the Indo-Pacific region, spanning the south of Japan, the east of India, the Indo-Pacific north of Australia, South Korea, the South China Sea, and Indonesia. The main habitats of *L. canarium* are the muddy sand bottom, coral sand bottom, and subtidal zone [1,2].

*L. canarium* is often found in sandy seagrass beds, particularly the seagrass belonging to the genus *Halophila*. Studies analyzing its stomach contents have reported that it cannot directly eat seaweeds because of its radula shape, and its major food sources are microalgae, filamentous algae, and diatoms present on seaweeds and seaweed detritus [1,2,3,4]. Similar ingestion patterns were found in *Conomurex luhuanus*, a close relative of *L. canarium*. According to the analysis of its stomach contents, *L. canarium* was reported to feed on tongue-grated particles together with grit and debris [5]. In addition, the study reported that indigestible sand and debris accounted for approximately 75–90% of its stomach content, whereas algae and diatoms accounting for only 7–15% of its stomach content that would be its potential sources of energy [5].

*L. canarium* has a high economic value and is one of the most vital fishery species and staple foods of residents in many coastal areas of Southeast Asia [6]. Currently, the fishing of *L. canarium* is still completely dependent on natural replenishment without aquaculture. However, in recent years, the number of *L. canarium* has been declining because of overfishing and environmental damage. Therefore, in many countries, resource surveys and management are underway for *L. canarium* [5,6,7,8,9]. Because they are increasingly scarce, *L. canarium* is increasingly valuable. The retail price of *L. canarium* in Taiwan can reach US $10–15/kg.

The aquaculture of *L. canarium* is the best approach to preventing its continual overfishing and preserving the natural environment. However, the main food sources of *L. canarium* are yet to be determined, and suitable techniques for its aquaculture have yet to be developed [1,2,6]. In our preliminary studies, we did not observe any active feeding behaviors from *L. canarium* fed by the artificial feed. Alternatively, the algae, biological detritus, and bottom mud were used as the feeds. In commercial farming, providing a large amount of nutritious organic detritus is difficult and expensive to manage [10,11]. 

Therefore, this study examined the most favorable diets and salinity tolerance for the growth of *L. canarium* from the larvae, newly metamorphosed, and 10 mm juvenile stages. In addition, we have developed a novel polyculture system with water flow to provide biological detritus and evaluated the feasibility and benefits of polyculture with other species. The findings of this study can reveal the potentiality of *L. canarium* in integrated multitrophic aquaculture (IMTA) and its implications for aquaculture and resource restoration.

## 2. Materials and Methods

### 2.1. Broodstock and Larval Rearing

All experiments were conducted in Gongliao Aqua Center, New Taipei City, Taiwan. Broodstocks of *L. canarium* were collected from the wild (Penghu island, Taiwan) and reared in a tank-based flow-through system (2500 L round fiberglass reinforced plastic (FRP) tanks). Broodstocks were fed once a day at 17:00–18:00, and leftover feed and dead individuals were removed before feeding. Commercial tilapia feed (24% protein) and sea cucumber feed (34% protein) was used in the preliminary diets test. The amount of daily feed was equal to 5% of body weight for *L. canarium.* However, we did not observe active feeding behavior. Diatoms and other biological detritus produced by feed may be their main source of nutrients.

**Figure 1:**
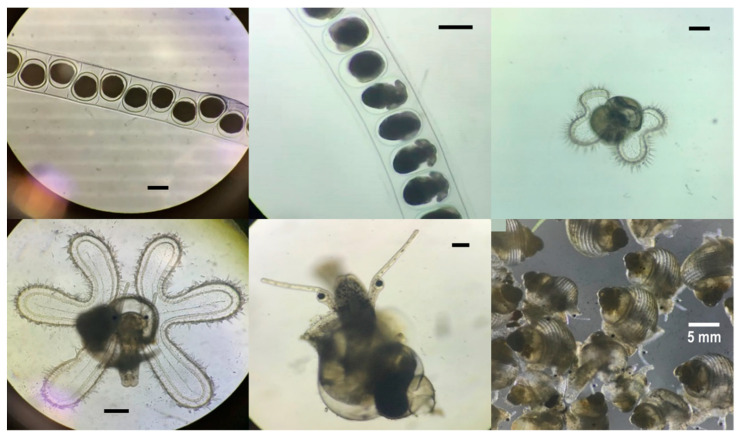
The development stages of *Laevistrombus canarium*. Egg capsules (**top left**); embryo (**top center**); 4-lobed veliger (top right); 6-lobed veliger (bottom left); newly metamorphosed juveniles (bottom center) (Scale bar = 100 µm); 10 mm juveniles (bottom right) (Scale bar = 5 mm).

During the breeding season (May to September), broodstocks (*n* > 250) were randomly mated and laid egg capsules at the bottom. Egg masses were collected and placed in plastic baskets with a mesh size of 0.5 cm and subsequently submerged in an adequately aerated hatching tank. Seawater was replenished daily until hatching. The rearing and culturing methods used were according to those reported by Cob et al. [7]. After hatching, swimming veligers were collected using a 50 μm mesh net and then transferred into 2500 L round FRP tanks at a concentration of 100 larvae/L. Natural seawater filtered using a 1 μm filter and mild aeration was used. The salinity and temperature of larval culture seawater were maintained at 34 ± 1% and 28 ± 1 °C, respectively; larval culture was maintained under 12:12 h light–dark conditions. Seawater was exchanged every 2 days with freshly filtered (using a 1 μm filter) natural seawater. *L. canarium* veliger larvae were fed the alga *Isochrysis galbana* and *Tetraselmis chui* at 1000 cells/mL once a day throughout the culture period. Fourteen days after hatching, the larvae of *L. canarium* developed to stage IV veliger that had a six-lobed velum that was considerably elongated with a finger-like appearance [7] (Figure 1). The individual with a well-developed proboscis and a dark green pigment at the later stage was subsequently moved into 2000 L FRP tanks for cultivation. Diatom-attached plastic plates were used to induce metamorphosis in seedlings and function as a part of the initial diet. Seedlings present at the sinking bottom were fed natural diatoms.

### 2.2. Salinity Tolerance

Salinity tolerance experiments for stage II veligers [7] and newly metamorphosed juveniles were conducted in 5 L beakers containing 3 L of seawater. The beakers had different salinity levels (35, 30, 25, 20, 15, and 10‰), and all experiments were performed in triplicate. A total of 300 veligers and 50 juveniles were placed in each beaker for 0–96 h and 0–30 days, respectively. The culture seawater was maintained at a temperature of 28 ± 1 °C under 12:12 h light–dark conditions. To enable the continual movement of larvae, all beakers were mildly aerated. At 0, 24, 48, 72, and 96 h of the experiment for veligers and at 0, 1, 2, 15, and 30 days for juveniles, each sample was examined under a dissecting microscope, and larvae were evaluated to be swimming or dead. Larvae that were lying at the bottom of the container but were capable of swimming if distributed were considered as being alive.

### 2.3. Diet Experiments

#### 2.3.1. Newly Metamorphosed Juveniles (1 mm)

**Figure 2:**
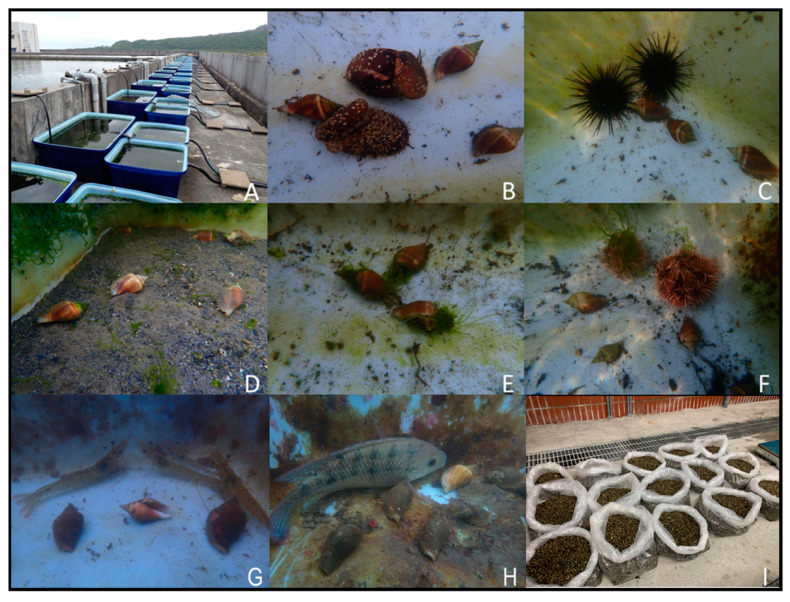
The experiment of juvenile *Laevistrombus canarium* cocultured outdoor with different species. (**A**) plastic tank: 60 × 45 × 90 cm; (**B**) abalone *Haliotis diversicolor*; (**C**) purple sea urchin *Anthocidaris crassispina*; (**D**) with sands; (**E**) control; (**F**) collector urchin *Tripneustes gratilla*; (**G**) shrimp *Litopenaeus vannamei*; (**H**) tilapia *Oreochromis mossambicus*; (**I**) mass production of juveniles in the Gongliao Aqua Center.

Diet experiments for newly metamorphosed juveniles were conducted in 5 L beakers containing 3 L of seawater. The groups were fed natural diatoms, sea cucumber feed (Omaga sea cucumber artificial feed; Chuan Kuan Enterprise, Kaohsiung, Taiwan), *Thalassiosira weissflogii* (TW1800; Reed Mariculture, Campbell, CA, USA), *Thalassiosira pseudonana* (TP1800; Reed Mariculture, Campbell, CA, USA), and kelp powder (Omaga seaweed powder; Chuan Kuan Enterprise, Kaohsiung, Taiwan). The nutrient composition of kelp powder was as follows: crude protein 12%, crude Ash 15%, moisture 11%, crude fat 0.4%, and crude fiber 14%. Nutrient composition of sea cucumber feed is as follows: crude protein 34%, crude Ash 15%, moisture 9%, crude fat 2.5%, crude fiber 4%, and HCl insoluble 1.8%. *T. weissflogii* and *T. pseudonana* were live and highly-concentrated commercial products (algae). They were stored at 4 °C and used up within four weeks. Additionally, an extra experiment was conducted in outdoor tanks (240 L, 90 × 60 × 45 cm) supplied with flow water from a 2,000,000 L concrete pool (Figure 2A). All experiments were performed in triplicate. The amount of daily feed was equal to 5% of the body weight of juveniles. A total of 100 juveniles were placed in each beaker for 30 days for each group. The salinity and temperature of culture seawater were maintained at 34 ± 1‰, and 28 ± 1 °C under 12:12 h light–dark conditions. To maintain an adequate level of dissolved oxygen, all beakers were mildly aerated. Seawater was exchanged every day with freshly filtered (using a 1 μm filter) natural seawater. At 0, 7, 14, 21, and 30 days into the experiment, each sample was examined under a dissecting microscope, and juveniles were examined to determine if they were alive or dead.

#### 2.3.2. Juveniles (10 mm)

Diet experiments for 10 mm juveniles were conducted in 10 L tanks containing 8 L of seawater. The groups were fed natural diatoms, sea cucumber feed, and kelp powder, and all experiments were performed in triplicate. The amount of daily feed was equal to the 5% of the body weight of juveniles. A total of 30 juveniles were placed in each tank for 56 days for each group. The experimental condition and an extra outdoor experiment were the same as Section 2.3.1 Newly metamorphosed juveniles. At 0, 7, 14, 21, 28, 35, 42, 48, and 56 days of the experiment, each sample was examined under a dissecting microscope. Juveniles were examined to determine whether they were alive or dead. While their body weight was regularly measured, their shell length (SL), width (SW), and depth (SD) were only measured on the final day of experiments (the 56th day) due to the limit of their small body size.

### 2.4. Indoor Polyculture Experiments

Polyculture experiments were performed using juveniles in 16 L tanks (30 × 55 × 10 cm) at 18 °C and 28 °C. The groups were either monocultured (cultured without sand as the control and cultured with sand) or cocultured with shrimp *Litopenaeus vannamei*, tilapia *Oreochromis mossambicus*, abalone *Haliotis diversicolor*, purple sea urchin *Anthocidaris crassispina*, and collector urchin *Tripneustes gratilla*. Clean and dry sand was used in the beginning. All experiments were performed in triplicate. A total of 30 juveniles (SL: 16.5 ± 1.2 mm and weight: 0.34 ± 0.08 g) were placed in each tank for 90 days for each group. The total body size of cocultured species was larger than that of *L. canarium* to ensure that it received adequate food sources (fecal matter from coculture species). The size of coculture species was 3 cm (shrimp), 5 cm (tilapia), 2 cm (abalone), 2 cm (purple sea urchin), and 2.5 cm (collector urchin). The amount of daily feed was equal to 5% of the body weight of the cocultured species. Commercial shrimp feed (50% protein) was used in shrimp coculture groups; commercial tilapia feed (24% protein) was used in tilapia coculture groups; and commercial sea cucumber feed (34% protein) was used in abalone, purple sea urchin, and collector urchin coculture groups. The salinity and temperature of culture seawater were maintained at 34 ± 1‰ and 18 or 28 ± 1 °C under a 12:12 h light–dark condition. To ensure an adequate level of dissolved oxygen, all tanks were mildly aerated. A recirculating aquaculture system was used, and 80% seawater was exchanged every 2 days with freshly filtered (using a 1 μm filter) natural seawater. At 0, 15, 30, 45, 60, 75, and 90 days into the experiment, each sample was examined by the naked eye to determine whether they were alive or dead. Various linear morphological characteristics of the shell were measured to a precision of 0.01 mm by using a digital vernier caliper. The measurements included SL, SW, and SD. All the procedures were conducted in accordance with ethical guidelines governing research using animals for in vivo experiments.

### 2.5. Outdoor Polyculture Experiments

Outdoor polyculture experiments for juveniles were conducted in 240-L tanks (90 × 60 × 45 cm) (Figure 2). The groups (monoculture and coculture) were the same as Section 2.4. Indoor polyculture experiments. A total of 30 juveniles (SL: 16.5 ± 1.2 mm and weight: 0.34 ± 0.08 g) were placed in each tank for 270 days for each group. The total body size of the coculture species was larger than that of *L. canarium* to ensure that. it received adequate food sources (feces from coculture species). The sizes of the coculture species and their feed were the same as Section 2.4. Indoor polyculture experiments. The flow-through system was used. The salinity of natural seawater was between 28 and 34‰, and its temperature ranged from 7 to 32 °C (the highest and lowest temperature recorded during September to June). To maintain an adequate level of dissolved oxygen, all tanks were mildly aerated. At 0, 30, 60, 90, 120, 150, 180, 210, 240, and 270 days into the experiment, each sample was measured as Section 2.4. Indoor polyculture experiments.

### 2.6. Statistical Analysis

The statistical analysis was conducted in SPSS 19 (IBM, Armonk, NY, USA), and the results are presented in terms of the mean  ±  standard deviation. All the data were verified to the normal distribution by Shapiro–Wilk test (*α*  =  0.05). The data were subjected to one-way analysis of variance (ANOVA) and Tukey’s honestly significant difference post hoc multiple range test (Tukey’s HSD) to examine differences between treatments.

## 3. Results

### 3.1. Salinity Tolerance

Table 1 lists the results of the tolerance of *L. canarium* (stage II veligers and newly metamorphosed juveniles) to salinity. The survival rate of the stage II veliger groups exposed to 25–35 salinities was 98–100% until the end of the experiment (not significant). The aforementioned three groups could normally metamorphose to veliger stage III (Figure 1). The survival rate of the groups exposed to below 20 salinities decreased to 80, 15, and 10% after 24 h, respectively, and all veligers died after 96 h. The vigor of living seedlings exposed to 15 and 10 salinities was poor, and only the cilia were observed to be moving (they swung limply and did not swim). The survival rate of the juvenile groups exposed to 35, 30, 25, and 20 salinities was 100% after 30 days (Table 1). The aforementioned four groups could grow normally (Figure 1). The survival rate of the group exposed to 15 salinities was 72% at 15 days and 47% at 30 days. Furthermore, the survival rate of the group exposed to 10 salinities decreased to 6% after 24 h, and all juveniles died after 48 h. The vigor of living juveniles exposed to 15 and 10 salinities was poor.

The tolerance and suitable salinity range of *L. canarium* become wide with the growth stages. Occasionally, the salinity is down to 15–20‰ after heavy rains. We could found a significant negative effect on veligers and moderate on newly metamorphosed juveniles. However, no abnormal behavior of the broodstock was observed.

### 3.2. Diet Experiments

#### 3.2.1. Newly Metamorphosed Juveniles (1 mm)

**Figure 3:**
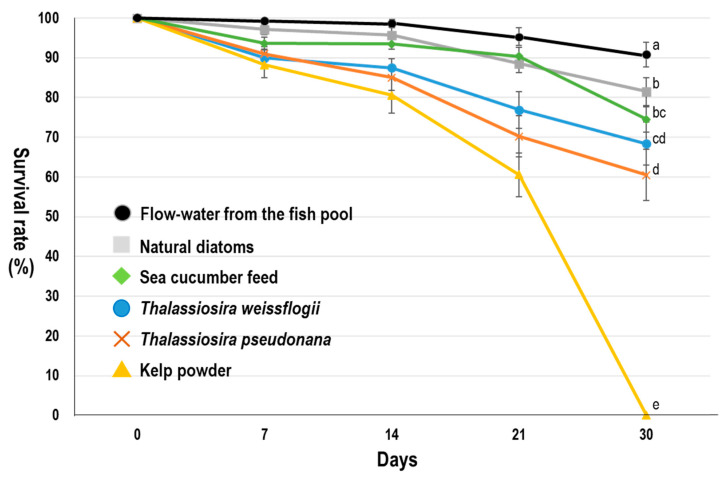
Survival rates of newly metamorphosed juvenile *Laevistrombus canarium* fed with different diets. The data of flow water from the fish pool were from outdoor tanks. We use 240 L tanks (90 × 60 × 45 cm) for juveniles and use flow-water from the 2000 tons fish pool (Figure 2A). Groups show significant differences by the 30th day (ANOVA; *p* < 0.001). Lowercase letters indicate significant differences (Tukey’s HSD; *p* < 0.05) of examined variables between diets. Bars present the mean ± standard deviation.

Figure 3 presents the survival rate of the newly metamorphosed juvenile *L. canarium* fed with different diets. The mean survival rates of groups fed with natural diatoms, sea cucumber feed, *T. weissflogii*, *T. pseudonana*, and kelp powder were 81.5, 74.5, 68.4, 60.5, and 0%, respectively, until the end of the experiment. The aforementioned four diet groups, except for the kelp powder group, could grow normally. The highest mean survival rate (90.5%) was observed in extra outdoor tanks with flow water from the fish pool. Although natural diatoms were also collected from the fish pool in the diet experiment, the flow water system can continuously provide diatoms and other biological detritus, and to get a better survival rate.

#### 3.2.2. Juveniles (10 mm)

**Figure 4:**
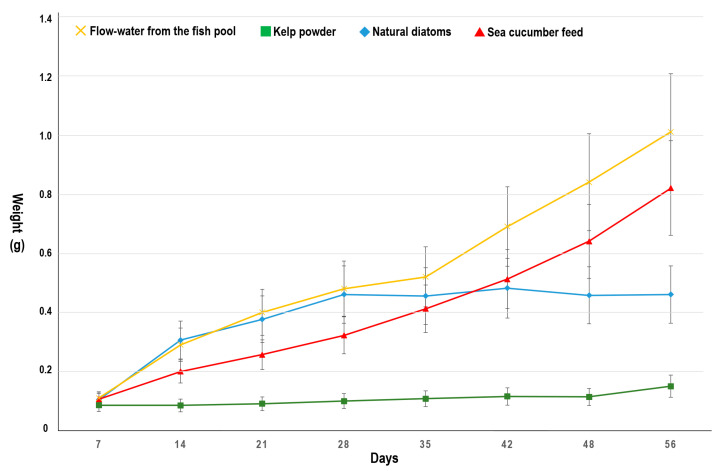
*Laevistrombus canarium*. Mean (± sd) weight of 10 mm juveniles at various sampling during 56 days of feeding on four diets (flow water, natural diatoms, sea cucumber feed, kelp powder).

Figure 4 and Table 2 show the weights of the 10 mm juvenile *L. canarium* fed with different diets and extra outdoor group. The final weights of groups fed with natural diatoms, sea cucumber feed, kelp powder, and flow-water were 0.46 ± 0.21, 0.82 ± 0.16, 0.15 ± 0.25 g, and 1.01 ± 0.51, respectively. The survival rates were 100% except for kelp powder (76%). The weight, SL, SW, and SD significantly differed among the groups (*p* < 0.05; Table 2). Natural diatoms are good in the first 28 days, however, their nutrition might not be enough for growth after 28 days ago. On the other hand, sea cucumber feed is not best in the first 28 days, however, their nutrition is enough for growth after 28 days ago. It is the same as Section 3.2.1. Newly metamorphosed juveniles, the highest survival rate and weight (100% and 1.01 ± 0.51) were observed in the extra outdoor tanks with flow water from the fish pool. It means Diatoms and biological detritus from the fish pool can provide enough diets with good nutrition for newly metamorphosed juveniles to at least 25 mm.

### 3.3. Indoor Polyculture Experiments

**Figure 5:**
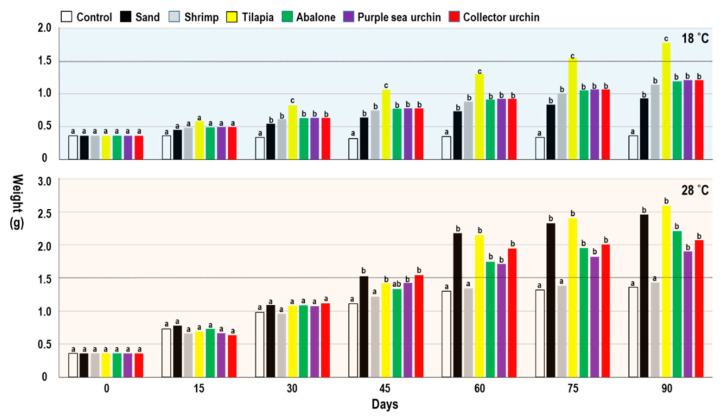
*Laevistrombus canarium*. Indoor polyculture experiments. Mean (± sd) weight of juveniles at various periods of coculture treatments (control, sand, cocultured with shrimp, tilapia, abalone, purple sea urchin, or collector urchin) at two temperature regimes (18, 28 °C). Different letters indicate statistically significant differences (Tukey’s HSD; *p* < 0.05) between coculture treatments at each sampling day (0 to 90th day post-initiation of the experiment).

Figure 5 and Table 3 present the weights of the juvenile *L. canarium* cocultured with different species at 18 and 28 °C. The weights of the groups cultured at 18 °C were between 0.36 ± 0.1 g (control, monoculture without sand) to 1.78 ± 0.32 g (coculture with tilapia) (Table 3). All groups significantly differed from the control group with respect to weight (*p* < 0.05; Table 3). The weights of the groups cultured at 28 °C were 1.36 ± 0.36 g (control, monoculture without sand) to 2.59 ± 0.73 g (coculture with tilapia) (Table 3). Coculture with tilapia showed the highest weights at 18 and 28 °C. Except for the group that was cocultured with shrimp, all groups significantly differed from the control group with respect to weight (*p* < 0.05; Table 3). The survival rate (%) of the groups cultured at 18 °C were 81 (control, monoculture without sand) to 99 (coculture with abalone) (Table 3). The survival rate (%) of the groups cultured at 28 °C was 95 (coculture with purple sea urchin) and 96 (coculture with purple sea urchin) to 100 (other groups) (Table 3). In indoor polyculture experiments, we found that (1) at high temperature; (2) with bottom sand; and (3) coculture with other organisms, there are obvious benefits in growth and survival rate. Although bottom sand is a significant benefit to *L. canarium*, coculture with purple sea urchin, collector urchin, and abalone can achieve similar results with bottom sand. It has the best performance when it is cocultured with tilapia. We observed the obvious feces deposited in the bottom when cocultured with tilapia, which is presumed to be beneficial to the feeding of *L. canarium*, thus directly improving the growth. Additionally, it was no significant benefit when cocultured with shrimp. We observed that the shrimp often disturbs the feeding of *L. canarium*. In the high-temperature group (28 °C), the shrimp is more active and directly disturbs, and competes with *L. canarium* to feed the biological detritus. Therefore, it directly affects the growth of *L. canarium*.

### 3.4. Outdoor Polyculture Experiments

**Figure 6:**
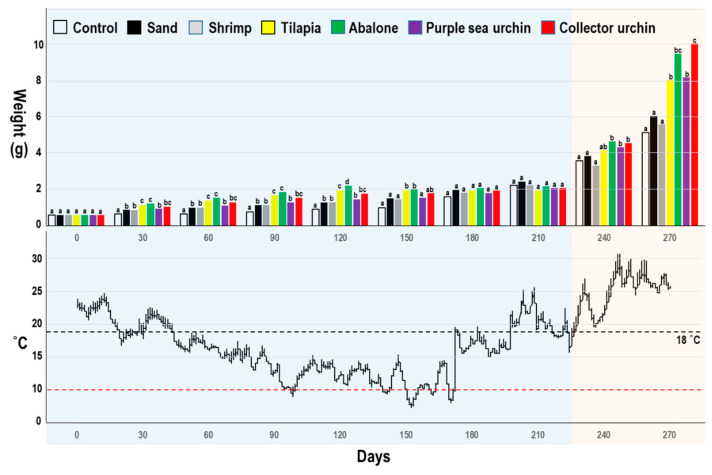
*Laevistrombus canarium*. Outdoor polyculture experiments. Mean (± sd) weight of juveniles at various periods (0 to 270 days post initiation of the experiment) coculture treatments (control, sand, cocultured with shrimp, tilapia, abalone, purple sea urchin, or collector urchin). Mean daily outdoors water temperature values during the experimental period. Different letters indicate statistically significant differences (Tukey’s HSD; *p* < 0.05) between coculture treatments at each sampling day (0 to the 270th day post-initiation of the experiment).

The outdoor polyculture experiment could be separated into the low-water-temperature period (0–210 days) and high-water-temperature period (210–270 days). The weights of the juvenile *L. canarium* cocultured outdoor with different species are shown in Figure 6 and Table 4. The weights were 5.18 g to 10.11 g (Table 4). Except for the group cocultured with shrimp, all coculture groups significantly differed from the control group with respect to weight (*p* < 0.05; Table 4). The survival rate (%) of the outdoor groups was 61 to 92 (Table 4). In outdoor polyculture experiments, we found some consistent results with indoor polyculture experiments. No significant difference among the groups was found in the low-temperature period (below 18 °C). 

## 4. Discussion

The food preferences of gastropods depend on the habitat and availability of a particular species as food [12,13,14]. Herbivorous gastropods mainly live on macroalgae, including green, red, and brown algae [15,16]. Several studies have reported that *L. canarium* is widely distributed in the seagrass bed ecosystem of Indian and Pacific regions [17,18,19,20]. Seagrass leaves provide a place for planktonic organisms to settle. When they settle on seagrass leaves, they are called epiphytes. Algae are the most abundant and diverse group to colonize seagrass leaves. Algal epiphytes substantially contribute to the primary productivity of the ecosystem (20–60%) and form the base of many food webs within seagrass communities. In field observations, *L. canarium* mainly grazed on epiphytes occurring on seagrass (46.67%), on the sediment surface (40%), and on macroalgae (13.33%) [3]. The food items found in the conch stomach included diatoms, detritus, foraminifera, seagrass and macroalgae fragments, sand particles, and shell fragments [3]. The three main types of food for *L. canarium* are diatoms, sand particles, and detritus [3]. In this study, commercial tilapia feed (24% protein) and sea cucumber feed (34% protein) were used in the preliminary diets test, however, we did not observe active feeding behavior. Consequently, it is more likely that diatoms and other biological detritus derived from feed could be their main source of nutrients. Therefore, polyculture groups grew faster than the monoculture group, which further implies that *L. canarium* might not directly benefit from the feed.

The food digestibility of adult *L. canarium* is between 55.21 and 74.75%, which is lower than that of other herbivorous gastropods [4,9]. The gastric content of *L. canarium* is 20–30% in volume, and the debris that cannot and can provide energy comprising 45–60 and 7–15% of gastric content, respectively. These findings indicate that the proportion of sand is higher in the stomach content of *L. canarium* [4,9]. In this study, the growth rate of the monoculture without sand group at the two temperatures was significantly lower than in that of the monoculture with sand group (Table 3). This finding indicated that *L. canarium* itself could not effectively cut and grind food by itself. The ingestion of sand helps in its digestion and absorption. It is worth mentioning that we found *L. canarium* cocultured with other organisms could grow well or even better than those without the bottom sand (Table 3 and Table 4). This means that the bottom sand could theoretically provide a good source of nutrients for digestion, but it is not critically necessary. Furthermore, the coculture of *L. canarium* without the substrates could be practically valuable since the accumulation of excessive food residues in the substrates could easily lead to death.

After metamorphosis, juvenile *L. canarium* becomes benthic grazers. It directly consumes algal epiphytes and cannot eat large seaweed alone [2]. The proportion of diatoms (44.21%) was the highest in its stomach, followed by those of sand (30.08%), detritus (15.6%), and seaweed fragments (8.55%) [4,9]. Powdered seaweeds have been used as nutrition sources for raising aquatic benthic animals (such as sea cucumber and mollusks) in hatcheries [21,22,23,24]. However, the group fed with kelp powder had the lowest survival and growth rates not only in the post-metamorphosis juvenile but also in 10 mm juvenile samples. This finding indicated that the kelp powder could not be effectively digested (high fiber content; 14% crude fiber) or provide complete nutrition to juveniles (low protein content; 12% crude protein) (Figure 3 and Figure 4). In the initial diet experiment (post-metamorphosis juvenile), the group fed with natural diatoms had a good growth rate in the first 30 days, similar to the flow-water method (Figure 3). For the 10 mm juvenile, the group fed with sea cucumber feed had a similar growth rate similar to the flow-water method. These findings indicate that natural diatoms were a favorable nutrition source in the first 30 days but did not provide adequate nutrition to *L. canarium* that were bigger in size (>10 mm; Figure 4). Sea cucumber feed was a favorable nutrition source in the later 30 days (Figure 4). A good feeding method is to use natural diatoms for the first 30 days before switching to sea cucumber feed. However, this study found the flow water method could increase the growth and survival rate of *L. canarium* (Figure 3 and Figure 4). Organic debris from the fish pool can provide complete development nutrition and can effectively form a commercial breeding method. We successfully used this flow-water to culture more than 300 thousand juveniles in a 200 tons pool in Aqua Center (Figure 2I).

This study proposes the polyculture and flow water method from a fish pool to provide the source of food for *L. canarium*, but it is necessary to pay attention to the temperature and salinity factors on the *L. canarium*. In tropical to subtropical regions, the temperature and salinity of land-based saltwater aquaculture are usually 15–30 °C and 15–35‰, respectively. Substantial salinity fluctuations due to heavy rains are common in saltwater ponds [25,26]. Salinity often plays a major role in determining the distribution of many marine organisms, primarily because of its effects on many physiological and behavioral processes [27]. Temperature is a factor that limits the distribution of marine animals, affects their activity levels, and disrupts their energy balance [28]. Most aquatic organisms cannot adjust their body temperature according to the surrounding environment; thus, most physiological and biochemical processes depend on the ambient temperature [29,30]. In our preliminary study, *L. canarium* was cultured in saltwater ponds throughout the year (at a temperature of 8–32 °C and a salinity level of 15–40‰). 

The growth rate of juvenile *L. canarium* is mainly dependent on the water temperature. In long-term outdoor experiments, we observed that the growth rate of *L. canarium* gradually increased with an increase in the water temperature (Figure 6). Even when the temperature is lower, the *L. canarium* shows lower growth and higher mortality, but they are still in the range that juvenile *L. canarium* can survive. Although northern Taiwan is a subtropical region, the water temperature of the land-based tanks in winter is sometimes less than 10 °C. When we check the survival on the 150th day and the 180th day, we found that a water temperature of less than 10 °C might cause death (Figure 6). At a low water temperature, the survival and growth rates of *L. canarium* were significantly decreased. Hassan et al. [4] reported that the most favorable temperature range for feeding and digestion is 26–30 °C for sub-adults *L. canarium*. The order of feeding and digestion rates was 26, 30, 34, and 22 °C, and these rates were significantly higher at 34 °C than at 22 °C. These results reflect the natural habitat of *L. canarium* and its adaptability to high temperatures (28–30 °C) [7].

We examined the tolerance of early life stages of *L. canarium* to low salinity levels and found no significant difference among the survival rates of pelagic larvae exposed to salinity levels of 25, 30, and 35‰. Similar results were found in benthic juveniles; however, they exhibited increased tolerance to lower salinity levels (20‰) (Table 1). The suitable salinity level for juvenile dog conch is between 20 and 35‰, indicating a wide range that is similar or better than those for sea urchins and sea cucumbers [31,32,33,34]. It means that the flow-water method from aquaculture ponds to provide early food sources for *L. canarium* has practical potential, even the salinity of the land-based ponds being more susceptible than natural seawater.

In this study, we found sand could be used for providing growth substrate of biofilms and diatoms (cultured with sand), or feed on natural sedimentary debris and diatoms (cultured without sand as the control) but also on feces and residual bait from other cultured organisms (cocultured). Although we did not analyze stomach content or stable isotopes, *L. canarium* grows better when they coculture with other organisms (Figure 5 and Figure 6). When the water temperature was 28 °C, the growth rates of the groups of polyculture (except whiteleg shrimp) were higher than the monoculture groups with sand. These findings indicated that fecal and residual nutrients from the aforementioned species could be effectively utilized by *L. canarium* even when sand is absent (Figure 5 and Figure 6). Although small abalone, collector urchin, and purple sea urchin are benthic species, they exert no negative effects on polyculture with *L. canarium* (Figure 5 and Figure 6). In addition, whiteleg shrimp affected *L. canarium* negatively, reducing their growth rate (Table 3 and Table 4). One possible reason is that *L. canarium* feeds on biofilms as a grazer and has competition with the shrimps; the shrimp often disturbs the feeding of *L. canarium*, which is why the growth rates in this combination were significantly worse than in the others. In the later period, we found whiteleg shrimp grow faster than *L. canarium*, and the large size shrimp even cause many *L. canarium* deaths (Table 4). While this work has shown that shrimp might not be a suitable species to coculture with *L. canarium*, it is still likely to coculture *L. canarium* with shrimps in different pools or net cages. Further study is necessary to show the high potentials by using organic debris from shrimp pools (e.g., biofloc method) [35].

In commercial aquaculture, approximately 50–70% of the operational cost is spent on feed [36,37]. Thus, how to reusing residual feed and organic debris and generating less wastewater is critically important [10,38,39]. In IMTA, one kind of polyculture method, the uneaten feed and waste of one species are collected and converted into feed, fertilizer, and energy for another species [40,41,42,43,44]. For example, large organic solids are generated from feed waste or feces and can be consumed by deposit feeders such as sea urchins and sea cucumbers [32,38,39]. Although sea urchins and sea cucumbers are favorable IMTA species, they have mainly been used in marine cages. Some land-based saltwater aquaculture techniques (e.g., earth ponds) are unsuitable for sea urchins and sea cucumbers [31,32,37]. In this study, the polyculture and the water-flow method show high benefits in *L. canarium* production. The high growth rate and adaption of *L. canarium* are advantageous in tropical and subtropical IMTA. The findings revealed that *L. canarium* could efficiently adapt to different environmental conditions and thus can be a favorable IMTA species. We propose *L. canarium* as a new favorable IMTA species that can be coculture with abalone and sea urchins in seawater or used in land-based saltwater aquaculture (e.g., saltwater tilapia).

## 5. Conclusions

This study examined the effectiveness of different diets to grow larvae and juveniles of dog conch *Laevistrombus canarium* and also tested the feasibility of its co-culture with other marine species. We developed a novel polyculture and water-flow method for mass production of this species. The suitable salinity range and diets were determined in the early life stage of *L. canarium*. We found that the polyculture system without substrates is possible for juveniles. In addition, water temperature is the most crucial factor for its growth and survival since it could die if the water is colder than 10 °C. Our study reports that *L. canarium* is highly promising in IMTA cultivation.

## Figures and Tables

**Table 1:** Survival rates (%) of veligers and newly metamorphosed juveniles of *Laevistrombus canarium* exposed to different salinity levels.

**Veligers**	**Salinity**	**0**	**24 h**	**48 h**	**72 h**	**96 h**
	35	100	100 ^a^	100 ^a^	100 ^a^	100 ^a^
	30	100	100 ^a^	100 ^a^	100 ^a^	100 ^a^
	25	100	100 ^a^	100 ^a^	99 ^a^	98 ^a^
	20	100	80 ^b^	48 ^b^	19 ^b^	0 ^b^
	15	100	15 ^c^	0 ^c^	0 ^c^	0 ^b^
	10	100	10 ^d^	0 ^c^	0 ^c^	0 ^b^
**Juvenile**	**salinity**	**0**	**24 h**	**48 h**	**Day 15**	**Day 30**
	35	100	100 ^a^	100 ^a^	100 ^a^	100 ^a^
	30	100	100 ^a^	100 ^a^	100 ^a^	100 ^a^
	25	100	100 ^a^	100 ^a^	100 ^a^	100 ^a^
	20	100	100 ^a^	100 ^a^	100 ^a^	100 ^a^
	15	100	100 ^a^	100 ^a^	72 ^b^	47 ^b^
	10	100	6 ^b^	0 ^b^	0 ^c^	0 ^c^

Veligers: stage II veligers according to Cob et al. [7]. ^a, b, c, d^ Values in rows with different lowercase letters indicate significant differences (Tukey’s HSD; *p* < 0.05).

**Table 2:** *Laevistrombus canarium*. Mean (±sd) values of weight, shell length, shell width, shell depth, survival rate, and daily growth rate of 10-mm juveniles after 56 days of feeding on four diets (flow water, natural diatoms, sea cucumber feed, kelp powder).

Parameter	Flow-Water	Natural Diatoms	Sea Cucumber Feed	Kelp Powder	*p*
SL (mm)	25.65 ± 4.75 ^a^	18.09 ± 2.10 ^b^	20.89 ± 3.79 ^c^	11.73 ± 2.35 ^d^	***
SW (mm)	12.49 ± 2.51 ^a^	8.76 ± 1.06 ^b^	10.17 ± 2.01 ^c^	5.51 ± 1.21 ^d^	***
SD (mm)	9.91 ± 1.88 ^a^	6.99 ± 0.80 ^b^	8.06 ± 1.49 ^c^	4.55 ± 0.95 ^d^	***
Weight (g)	1.01 ± 0.51 ^a^	0.46 ± 0.21 ^b^	0.82 ± 0.16 ^c^	0.15 ± 0.25 ^d^	***
Daily growth (g)	0.02	0.01	0.01	0	-
Survival rate (%)	100	100	100	76	-

^a,b,c,d^ Lowercase letters indicate significant differences (Tukey’s HSD; *p* < 0.01) of examined variables between diets. Daily growth: (mean final weight–mean initial weight)/56 days. *** ANOVA; *p* < 0.001.

**Table 3:** *Laevistrombus canarium*. Mean (±sd) of weight, shell length, shell width, shell depth, survival rate, and daily growth rate of juveniles after 90 days of coculture treatments (control, sand, cocultured with shrimp, tilapia, abalone, purple sea urchin, or collector urchin) at two temperature regimes (18, 28 °C).

**18 °C**	**Control**	**Sand**	**Shrimp**	**Tilapia**	**Abalone**	**PU**	**CU**	***p***
SL (mm)	18.17 ± 1.76 ^a^	18.00 ± 2.86 ^a^	18.77 ± 2.32 ^a^	22.54 ± 2.47 ^b^	20.09 ± 1.42 ^c^	20.48 ± 1.58 ^c^	20.11 ± 1.46 ^c^	***
SW (mm)	8.85 ± 0.85 ^a^	9.10 ± 1.67 ^ab^	9.66 ± 1.26 ^c^	11.71 ± 1.22 ^d^	9.48 ± 0.65 ^bc^	10.03 ± 0.90 ^cd^	9.37 ± 0.91 ^cb^	***
SD (mm)	6.81 ± 0.66 ^a^	7.41 ± 1.32 ^b^	8.08 ± 1.07 ^c^	9.06 ± 1.13 ^d^	7.40 ± 0.41 ^e^	7.48 ± 0.69 ^e^	7.78 ± 0.65 ^ce^	***
Weight (g)	0.36 ± 0.1 ^a^	0.93 ± 0.4 ^b^	1.14 ± 0.42 ^c^	1.78 ± 0.32 ^d^	1.19 ± 0.12 ^c^	1.21 ± 0.28 ^c^	1.21 ± 0.34 ^c^	***
Daily growth (g)	0.00	0.01	0.01	0.02	0.01	0.01	0.01	-
Survival rate (%)	81	88	96	92	99	95	96	-
**28** **°C**	**Control**	**Sand**	**Shrimp**	**Tilapia**	**Abalone**	**PU**	**CU**	***p***
SL (mm)	25.75 ± 2.49 ^a^	30.45 ± 4.01 ^b^	25.57 ± 3.52 ^a^	30.52 ± 4.11 ^b^	29.95 ± 2.76 ^bc^	28.37 ± 2.32 ^d^	28.97 ± 2.23 ^cd^	***
SW (mm)	13.13 ± 1.27 ^a^	15.75 ± 2.24 ^b^	12.93 ± 1.80 ^a^	15.67 ± 2.16 ^bc^	15.74 ± 1.20 ^bc^	14.61 ± 1.32 ^d^	14.97 ± 1.21 ^cd^	***
SD (mm)	10.46 ± 1.05 ^a^	12.5 ± 1.86 ^b^	10.28 ± 1.48 ^a^	12.51 ± 1.88 ^b^	12.35 ± 0.78 ^bc^	11.49 ± 1.06 ^d^	11.94 ± 1.01 ^bcd^	***
Weight (g)	1.36 ± 0.36 ^a^	2.46 ± 0.59 ^b^	1.43 ± 0.61 ^a^	2.59 ± 0.73 ^b^	2.21 ± 0.53 ^c^	1.9 ± 0.44 ^d^	2.07 ± 0.58 ^cd^	***
Daily growth (g)	0.01	0.02	0.01	0.03	0.02	0.02	0.02	-
Survival rate (%)	100	100	100	100	100	95	96	-

^a,b,c,d,e^ Lowercase letters indicate significant differences (Tukey’s HSD; *p* < 0.05) between coculture treatments on the 90th day post-initiation of the experiment. Daily growth: (mean final weight–mean initial weight)/90 days). *** ANOVA; *p* < 0.001.

**Table 4:** *Laevistrombus canarium*. Mean (±sd) of weight, shell length, shell width, shell depth, survival rate, and daily growth rate of juveniles after 270 days of coculture treatments (control, sand, cocultured with shrimp, tilapia, abalone, purple sea urchin, or collector urchin) at outdoors water temperature.

Parameter	Control	Sand	Shrimp	Tilapia	Abalone	PU	CU	*p*
SL (mm)	40.70 ± 6.05 ^a^	42.89 ± 4.23 ^b^	39.97 ± 3.93 ^a^	47.49 ± 3.95 ^c^	49.42 ± 3.38 ^cd^	48.35 ± 3.73 ^c^	50.90 ± 3.34 ^d^	***
SW (mm)	20.24 ± 3.38 ^a^	22.07 ± 2.31 ^b^	20.61 ± 2.34 ^ab^	24.08 ± 3.05 ^c^	25.68 ± 3.27 ^d^	24.53 ± 3.01 ^cd^	27.28 ± 3.44 ^e^	***
SD (mm)	16.45 ± 2.49 ^a^	17.75 ± 1.94 ^b^	16.75 ± 1.93 ^a^	19.50 ± 2.36 ^c^	20.31 ± 1.71 ^cd^	19.79 ± 1.64 ^c^	21.06 ± 1.72 ^d^	***
Weight (g)	5.18 ± 2.57 ^a^	6.10 ± 2.31 ^a^	5.63 ± 1.82 ^a^	8.09 ± 2.50 ^b^	9.54 ± 2.39 ^c^	8.23 ± 1.92 ^b^	10.11 ± 2.28 ^c^	***
Daily growth (g)	0.02	0.03	0.02	0.03	0.04	0.03	0.04	-
Survival rate (%)	76	92	61	86	84	82	88	-

^a,b,c,d,e^ Lowercase letters indicate significant differences (Tukey’s HSD; *p* < 0.05) between coculture treatments at 270th day post initiation of the outdoor polyculture experiment. Daily growth: (mean final weight–mean initial weight)/270 days). *** ANOVA; *p* < 0.001.

## Data Availability

The data presented in this study are available on request from the corresponding author.
